# Factors influencing dialysis withdrawal: a scoping review

**DOI:** 10.1186/s12882-018-0894-5

**Published:** 2018-04-24

**Authors:** Hammad Ali Qazi, Helen Chen, Meng Zhu

**Affiliations:** School of Public Health and Health Systems, Faculty of Applied Health Sciences, University of Waterloo, Waterloo, ON Canada

**Keywords:** Dialysis, Hemodialysis, Peritoneal dialysis, Dialysis withdrawal, Dialysis discontinuation, Dialysis attrition, Scoping review

## Abstract

**Background:**

Research on factors associated with dialysis withdrawal is scarce. This study examined the predictors that might influence rate of dialysis withdrawal. Existing literature is summarized, analyzed and synthesized to identify gaps in the literature with regard to the factors associated with dialysis withdrawal.

**Methods:**

This scoping review used a systematic search to synthesize research findings related to dialysis withdrawal and identified gaps in the literature. The search strategy was developed and applied using PubMed, EMBASE and CINHAL databases. The selection criteria included articles written in English and published between 1997 and 2016 that examined dialysis withdrawal and associated factors in patients with any modality of renal dialysis.. Case reports and studies only including renal transplant patients were excluded. Fifteen articles were selected in accordance with these selection criteria.

**Results:**

The literature review revealed a scarcity of research on dialysis withdrawal and associated factors. Furthermore, the study findings were inconsistent and inconclusive. Authors have defined dialysis withdrawal in terms of dialysis discontinuation, withholding, death, withdrawal, treatment refusal/cessation, or technique failure. Authors have selected homogeneous patient population on either hemodialysis (HD) or peritoneal dialysis (PD) patients, thus making comparisons of studies and generalization of findings difficult.

**Conclusion:**

Future studies should explore the influence of both HD and PD on patient-elected dialysis withdrawal using a large a priori calculated sample size.

**Electronic supplementary material:**

The online version of this article (10.1186/s12882-018-0894-5) contains supplementary material, which is available to authorized users.

## Background

Chronic kidney disease (CKD) is the gradual loss of renal function over a period of months or years and is classified into five stages based on the measurement of estimated glomerular filtration rate (eGFR) [1, 2]. End stage renal disease (ESRD), or CKD stage 5, represents the most severe form of renal function, is characterized by an eGFR of < 15 mL/min per 1.73 m^2^ and requires maintenance dialysis or renal transplantation [1, 2]. The prevalence of both CKD and dialysis is increasing globally, mainly because of long-term survival rates [1, 2]. There are nearly 700,000, 120,000 and 135,000 people with CKD stage 5 in the United States (US), United Kingdom (UK), and Europe, respectively [3, 4]. The prevalence of ESRD in Saudi Arabia ranges from 5.7%–6% [5, 6] and 6% in Australia [6]. Furthermore, from 2006 to 2012 Canada had the third highest ESRD incident and prevalence rates after the US and Japan [7].

It is estimated that approximately 11,200 patients in Ontario are on dialysis with 76.3% on in center hemodialysis (HD), 18.1% on peritoneal dialysis (PD), and 5.6% undergoing home hemodialysis (HHD) [8]. Despite the importance of dialysis for patients in CKD stage 5, authors have found a significant rate of dialysis withdrawal (DW) ranging from 8% to 31% [8–11]. Dialysis attrition as a result of discontinuation or withholding is one of the leading causes of death (12%–26%) in ESRD patients in the US and Canada [12]. However, in European countries dialysis withdrawal, withholding, or discontinuation rates are lower in comparison and responsible for only 2% to 7% of all causes of deaths [12, 13].

There are many factors associated with dialysis withdrawal. Gesert et al. [14] have found a higher dialysis withdrawal rate in women versus men (26.3% versus 23.0%), older age versus younger age (29.83% versus 18.14%), and white versus black people (29.5% versus 14.7%). Factors such as diabetes-induced ESRD and renovascular disease were associated with an elevated withdrawal rate (hazards ratio [HR] = 1.58 and HR = 1.26, respectively) [10]. Additionally, having a body mass index (BMI) less than 18.5 kg/m (HR = 1.37) has been associated with increased rates of withdrawal [10]. Type of dialysis (PD or HHD), comorbid conditions such as diabetes and cardiac diseases, blood and serum markers such as albumin, phosphate, and hemoglobin levels have been shown to be associated with dialysis withdrawal [9–12, 15–19]. However, some studies have shown insignificant associations between gender, BMI, socioeconomic predictors, comorbidities, aetiology of renal disease, albumin and creatinine, and types and duration of dialysis with dialysis withdrawal [9–12, 16–20].

The scarcity of literature and the overall inconsistent and inconclusive findings warrant an in-depth exploration of the predictors that might influence rate of dialysis withdrawal and to identify gaps in the literature with regard to the factors associated with dialysis withdrawal, in which original research is needed. A scoping review was performed with the aim of exploring the literature in order to identify the factors that can influence dialysis withdrawal (the articulation of the specific research question formed a part of the scoping review and hence will be included in the methodology section of the review). This scoping review will provide a better understanding of the factors and their association in relation to withdrawal from dialysis. This understanding will help to improve clinical decision making by identifying patients who have higher risks of dialysis withdrawal, providing solutions, removing barriers, and facilitating the participation of patients in the decision making. In addition, the current review will help to collate, summarize, and report the research findings by identifying gaps and drawing conclusions from the existing literature [21].

## Methods

Scoping reviews are conducted to identify gaps and to explore areas in which research has been limited [21]. Scoping reviews also help to create a rich database of literature that can serve as a foundation for more detailed reviews. Scoping reviews follow systematic reviews by using rigorous and transparent methods for data collection (a systematic search of the relevant literature based on predefined selection criteria) [21]. Data charting, collating and summarizing the results from scoping reviews also enhance reliability and the potential for replication [22]. Scoping reviews focus on the studies’ research findings and not on how these findings were obtained [23].

A scoping review typically follows the following steps [21].

- Identifying the research question and searching for relevant studies
- Selecting the studies based on pre-defined selection criteria
- Charting the data, and
- Collating, summarizing, and reporting the results

### Identifying the research question and searching the relevant studies

The research question of this review was: “What are the factors associated with dialysis withdrawal in chronic dialysis patients?” The search strategy focused on two components: 1.) dialysis and 2.) dialysis withdrawal. Each component was later classified into relevant synonyms, key words, and MeSH terms, and a systematic search strategy was developed and applied using PubMed, EMBASE and CINHAL databases according to the advice of an experienced librarian specializing in Health Sciences at the University. The grey literature was not included in the search strategy because the authors believe most of the literature related to the objectives of this scoping review are published in the peer-reviewed journals thus contained in the databases. Similar strategy of excluding the grey literature is also used in other reviews on the topic of dialysis and ESRD [24–27]. The systematic search strategy is shown in Appendix (Additional files 1, 2 and 3).

### Selection of studies based on pre-defined selection criteria

To be included in this review, a study had to meet all of the following criteria

1. Examined dialysis attrition, withdrawal, discontinuation, stopping, withholding, cessation or refusal and associated factors such as age, gender, patient characteristics, diseases and comorbidities, dialysis indicators and blood and serum markers.
2. Included patients with any modality of renal dialysis such as home, facility, hemodialysis, or peritoneal dialysis.
3. Written in English.
4. Published from 1st January 1997 to 31st December 2016.

The exclusion criteria consisted of two parameters:

1. Studies only included renal-transplant patients.
2. Case series or case reports.

**Fig. 1:**
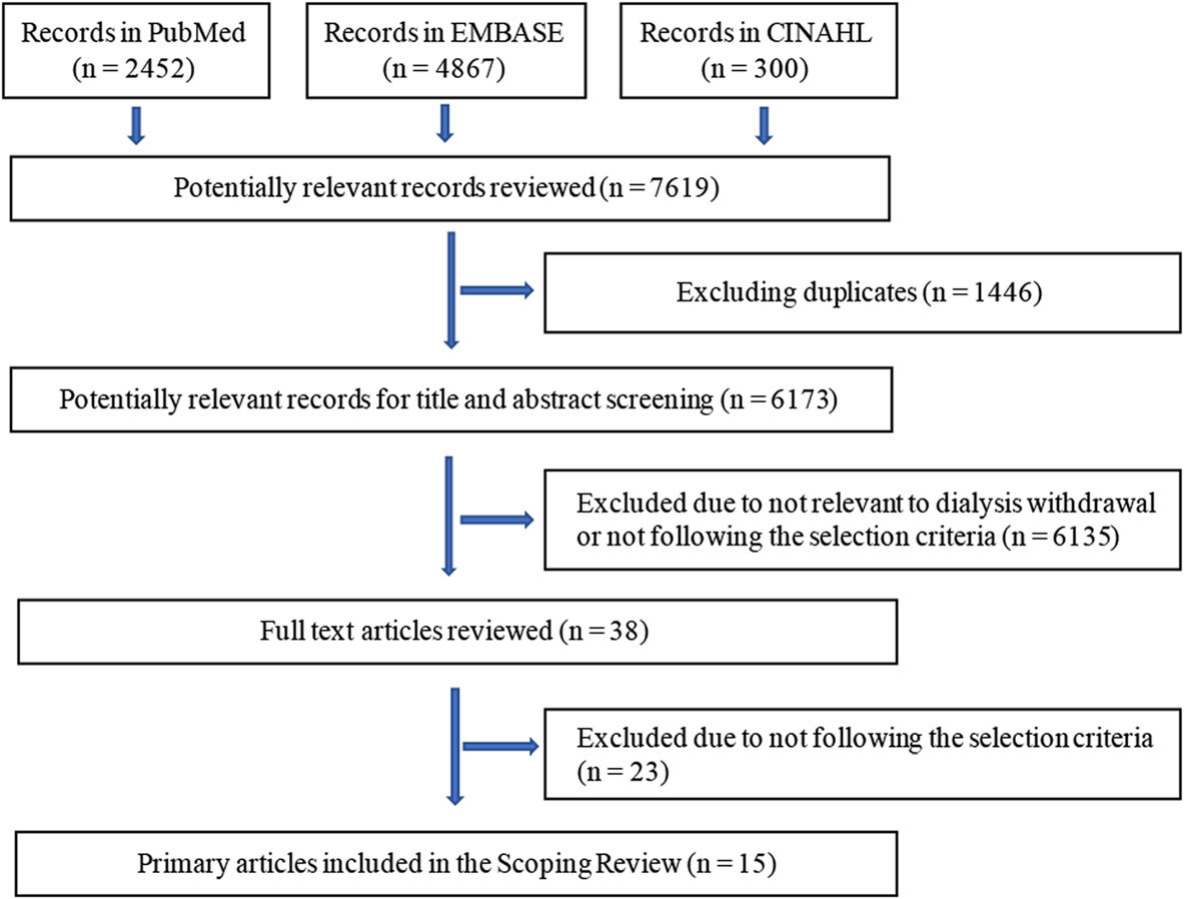
Systematic Selection of Records: Flow Diagram

The research strategy, selection criteria, and assessment of the studies were discussed with experts in the field. The titles and abstracts of the relevant articles identified by the systematic search strategies were used to categorize the articles as “included”, “excluded”, or “unsure” by each researcher independently based on the selection criteria. The full text of the “uncertain articles” were read to determine whether they should be included in the current review based on the selection criteria. Any disagreement in study selection between the researchers were resolved through discussion. The findings were recorded from the full text of the articles. Of the articles identified by the search strategy, only 15 met the selection criteria as described in Fig. 1.

### Data charting

A chart approach, based on a descriptive-analytical method, was used to synthesize and interpret the quantitative and qualitative data collected from the selected studies [21]. Following this approach, the authors’ names, year of publication, study setting, objectives of the study, study design, methods, and study results were recorded if available within a study. In order to extract the study results from the reviewed studies, the result sections from each of the full text articles were carefully read to identify statements pertaining to the predictors and variables related to dialysis withdrawal. References to the results of other studies in the discussion sections of the reviewed articles were excluded [28]. These statements, along with information related to the above variables, were entered on a separate form for each study. The summaries of the reviewed articles are provided in Table 1.

**Table 1:** Summary of Selected Articles (*N* = 15)

First Author	Year of Publication	Setting	Total Sample Size	Number of DW	Study Design	Factors associated with DW						
						Demographic factors	Renal causes	Health behaviors	Physiologic indicators	Comorbidities	Dialysis indicators	Others
Seshasai, RK [8]	2016	US	2840	25	Retrospective	√	NA	√	NA	√	√	√
Chan, HW [9]	2012	Australia/New Zealand	24,884	3.5–13.5	Retrospective	√	√	NA	NA	√	√	√
Mizuno, M [11]	2011	Japan	561	31	Retrospective	NA	NA	NA	NA	√	√	√
Ellwood, AD [10]	2013	Canada	46,181	1.5–3/100 patients	Retrospective	√	√	√	√	√	√	√
Workeneh, B [19]	2015	US	127	NA	Retrospective	NA	√	NA	NA	√	√	NA
Hazama, T [16]	2014	Japan	28	NA	Prospective	√	NA	√	√	√	√	√
Remon-Rodriguez, C [18]	2014	Spain	NA	NA	Retrospective	√	NA	NA	NA	√	√	√
McDade-Montez, EA [17]	2006	US	256	NA	Prospective	√	NA	NA	√	√	√	NA
Fissell, RB [31]	2005	Multiple	8615	NA	Retrospective	√	√	√	NA	√	√	√
Birmele, B [12]	2004	France	1436	NA	Retrospective	√	√	√	NA	√	√	√
Koc, Y [30]	2011	Turkey	61	48	Retrospective	NA	NA	NA	NA	NA	NA	√
Ashby, M [29]	2005	Australia	16	4	Qualitative	NA	NA	NA	NA	NA	NA	√
Chan, C [15]	2007	China	107	12	Retrospective	NA	NA	NA	NA	NA	NA	√
Moist, LM [20]	2008	Multiple	20,976	NA	Prospective	NA	NA	NA	NA	NA	NA	NA
Urban, AK [32]	2013	Australia	33	101	Retrospective	√	NA	NA	NA	√	√	√

### Collating, summarizing, and reporting the results

The collated information was initially presented as a description of the selected studies. It included a description of the numerical data and other collected information obtained from each of the quantitative and qualitative studies, including the authors’ names, study design, setting, publication year, and sample characteristics [21]. A thematic analysis was performed on the extracted statements identified from the results section of each full-text article. These statements were read several times to identify common themes related to dialysis withdrawal. The identified themes were discussed with field experts to minimize researcher bias.

## Results

The study characteristics such as type of studies, settings and publication are summarized by the number of studies (n) in Table 2. The maximum and minimum sample size, withdrawal rates of all included studies and identified themes and are summarized by the number of studies (n) are also included.

**Table 2:** Descriptive results summary

Items	Summary
*Type of Studies*	*N = 15*
Retrospective Study	*n* = 11
Prospective Study	*n* = 3
Qualitative Study	*n* = 1
*Setting*	*N = 15*
US	*n* = 5
Japan	*n* = 4
Australia	*n* = 4
Spain	*n* = 3
France	*n* = 3
Germany	*n* = 2
UK	*n* = 2
Italy	*n* = 2
Canada	*n* = 2
New Zealand	*n* = 2
Turkey	*n* = 1
China	*n* = 1
Belgium	*n* = 1
Sweden	*n* = 1
*Year of Publication*	*N = 15*
2004	*n* = 1
2005	*n* = 2
2006	*n* = 1
2007	*n* = 1
2008	*n* = 1
2011	*n* = 2
2012	*n* = 1
2013	*n* = 2
2014	*n* = 2
2015	*n* = 1
2016	*n* = 1
*Range of Sample size in studies*	
Minimum	16
Maximum	46,181
	*Dialysis Withdrawal Percentage*
Minimum	10%
Maximum	30%
*Themes*	*N = 15*
Demographic factors	*n* = 8
Renal causes	*n* = 5
Health behaviors	*n* = 5
Physiologic indicators	*n* = 3
Comorbidities	*n* = 10
Dialysis indicators	*n* = 11
Others	*n* = 13

### Dialysis withdrawal

The thematic analysis revealed that only a few authors (*n* = 3) used a specific definition of dialysis withdrawal. However, many authors (*n* = 12) used dialysis withdrawal for a combination of multiple reasons, such as overall dialysis discontinuation, technique failure, modality switch, clinician and patient-specific reasons, and death. Chan et al. (2007) [15] and Ashby et al. (2005) [29] define dialysis withdrawal as patients who discontinue dialysis therapy, whereas Koc et al. (2011) [30] define dialysis withdrawal as patients who withdrew or dropped out.

Authors using dialysis withdrawal defined as a combination of multiple reasons explored the association between predictors and dialysis withdrawal but did not perform a separate analysis for the relationship. Mizuno et al. (2011) [11] define dialysis withdrawal as patients who discontinue dialysis due to change in modality, death, complications, and social reasons. Hazama et al. (2014) [16] define dialysis withdrawal as technique failure, complications such as peritonitis, and preferences of the physicians, patients and family members. Seshasai et al. (2016) [8] define dialysis withdrawal as a discontinuation or change in dialysis modality within 60 days.

Chan et al. (2012), Birmele et al. (2004), McDade-Montez et al. (2006), Moist et al. (2008), and Fissell et al. (2005) [9, 12, 17, 20, 31] define dialysis withdrawal as all types of dialysis termination, including change in modality, death, and dialysis discontinuation by clinicians and patients. Ellwood et al. (2013) [10] and Workeneh et al. (2015) [19] define dialysis withdrawal as all types of discontinuation except for recovery patients and transplant and recovery patients, respectively. Remon-Rodriguez et al. (2014) [18] define dialysis withdrawal as discontinuation of peritoneal dialysis for any reason. Urban et al. (2013) [32] define dialysis withdrawal as elective withdrawal from dialysis by the patient, family, or medical team where continuing to prolong life by RRT was inappropriate or undesirable. Due to differences found in the definition of dialysis withdrawal among selected studies we were not able to synthesize and group our findings in the results section. However, we will discuss the similarities and differences of our findings with the literature in relation to definition of dialysis withdrawal in the discussion section.

### Influence of factors on dialysis withdrawal

i) **Influence of demographic factors on dialysis withdrawal:**
  Older age is positively (*n* = 4) associated with dialysis withdrawal when compared with younger age. Male gender (*n* = 2) was negatively associated with dialysis withdrawal when compared with females. White race was positively associated with dialysis withdrawal when compared with Asians, indigenous Canadians, and non-whites (*n* = 5). Demographic factors such as income, education, employment, marital status, and residence area were not associated with dialysis withdrawal [8–10, 12, 16, 17, 19, 31, 32].
ii) **Influence of renal disease aetiology on dialysis withdrawal:**
  A few authors (*n* = 3) explored the association between renal disease aetiology and dialysis withdrawal. Withdrawal from dialysis had no significant associations with renal diseases such as hypertension, diabetes, glomerulonephritis, renovascular disease (age < 75 years only), diabetic nephropathy, interstitial nephropathy, polycystic kidney disease, or glomerulopathy [10, 12, 19]. Only one study found that diabetes-induced ESRD and renovascular disease are associated with higher rates of dialysis withdrawal than glomerulonephritis [10].
iii) **Influence of health behavior on dialysis withdrawal:**
  Personal health and behavioral factors such as BMI (*n* = 1) and smoking/drug/alcohol use (*n* = 1) were positively related with dialysis withdrawal [8, 10]. Poor general health condition (*n* = 1) and dependency on others for daily activities (*n* = 1) were also significantly associated with attrition [12]. However, few authors did not find any association between BMI and alcohol/substance abuse during the last 12 months with dialysis withdrawal [16, 31].
iv) **Influence of physiologic indicators on dialysis withdrawal:**
  Blood and serum markers such as serum albumin (*n* = 1) and lower (≤ 3.31 g/dL) serum albumin (*n* = 1), decrease in serum creatinine (*n* = 1), and higher (> 27 pg/mL) dialysate vascular endothelial growth factor (*n* = 1) were significantly associated with dialysis withdrawal [10, 16, 17]. Other laboratory markers such as hemoglobin, serum potassium, uric acid and urea were inconsistently associated with dialysis withdrawal [10, 16, 17].
v) **Influence of comorbidities on dialysis withdrawal:**
  Authors found that comorbidities such as dementia (*n* = 3), diabetes (*n* = 2), cerebrovascular diseases (*n* = 1) and malignancy (*n* = 2) were associated with dialysis withdrawal [8–10, 12, 31]. Presence of comorbidities was also positively associated with withdrawal from dialysis. Authors found insignificant associations between heart diseases, vascular diseases stroke, lung diseases, cellulitis/gangrene, hepatitis B and C, and/or neurological diseases on dialysis withdrawal [8–12, 16–19, 31].
vi) **Influence of dialysis indicators on dialysis withdrawal:**
  Peritoneal dialysis (*n* = 1) was negatively associated with withdrawal [9]. However, a few authors also found no differences in relationship between dialysis modality and withdrawal [12]. Authors found that early initiation of dialysis (*n* = 1) and dialysis-associated pain (*n* = 1) were positively associated [10, 29], whereas, duration of dialysis, dialysis time and technique, weight gain, and dialysis adequacy measurements were not associated with dialysis withdrawal [8–12, 16–19, 31].
vii) **Influence of other individual factors on dialysis withdrawal:**
  Only a few authors have explored predictors such as late referral to a nephrologist (*n* = 2), community centre versus in-hospital dialysis (*n* = 1), and/or living alone as individual variables that influence dialysis withdrawal [9, 10]. Authors found that renal transplantation is associated with dialysis withdrawal [16, 30]. Similarly, another study showed that living alone was associated with dialysis withdrawal [32].

## Discussion

This review showed that many authors define dialysis withdrawal as generalized dialysis discontinuation for reasons including social factors, patient preference, the clinician’s opinion, modality change, transplant, recovery, and death. Few authors have used patient-selected dialysis withdrawal as a reason for dialysis discontinuation. The review identified a scarcity of literature on the relationship between individual factors and dialysis withdrawal. Moreover, there was conflicting evidence in few studies that explored the associations between dialysis withdrawal and demographics, renal diseases, health behaviour, comorbidities, physiological indicators, or dialysis factors.

### Influence of demographic factors on dialysis withdrawal

The relationship between demographic factors such as age, gender, race/ethnicity, residence, education, employment and marital status and dialysis withdrawal have been explored by only a few researchers in different geographical locations [8–10, 12, 16, 17, 19, 31, 32]. Older age was associated with higher rate of dialysis withdrawal. Discontinuation of dialysis was more frequent in patients ≥70 years old versus those < 70 years old (29.83% versus 18.14%, *p* < 0.001) [14]. Ellwood et al. (2013) found higher rates of withdrawal in patients aged ≥75 years as compared to patients aged < 75 years, and increasing age was significantly associated with dialysis withdrawal (HR, 1.81; 95% CI, 1.75–1.88) [10]. Similarly, Findlay et al. (2016) found older age was significantly associated with dialysis withdrawal [33]. Older age patients have multiple medical problems and comorbidities that worsen with increased duration of dialysis. These results suggest that this drastic change in physical and mental health leads to increased dialysis withdrawal and discontinuation of treatment in older populations [10, 12, 34]. Yet, Urban et al. (2013) [32] found insignificant differences with respect to age, gender, and living situation between elective dialysis withdrawal and non-withdrawal groups. This difference among studies was not related to the definition of dialysis withdrawal, as all the above studies used generalized (non-specific) discontinuation of dialysis [10, 12, 32, 34]. The difference could have been due to a small subsample (*n* = 10) in the study by Urban et al. [32] in which the number of people was insufficient to detect any differences among these factors.

Dialysis withdrawal also appears to vary with race and ethnicity. Patient-elected (based on patient decision) dialysis discontinuation was more frequent in whites than blacks (29.5% versus 14.7%, *p* < 0.001) or patients of other races (29.5% versus 19.2%, *p* < 0.001) [14]. Similarly, other authors have reported a higher rate of dialysis withdrawal in white people versus African Americans and Asians [14, 35–39]. This difference in dialysis withdrawal between different ethnicities is unclear although these findings highlight the role of social and cultural values in the decision to withdraw from or continue dialysis [9, 10]. One of the reasons for increased likelihood of dialysis withdrawal in whites may be related to more liberal values such as religious, societal, and cultural beliefs, which can have an influence in deciding to continue or withdrawal from dialysis [9, 40, 41]. The differences may also be more pronounced in a geographical setting with historical racial tensions and issues, such as in the US, where non-white races continue dialysis and have a lack of trust in healthcare settings because of inequalities in healthcare in comparison to white populations [13].

The association between dialysis withdrawal and gender is inconsistent and inconclusive. Few authors have shown that women are more likely to withdraw from dialysis than men and a higher dialysis withdrawal rate in women versus men (26.3% versus. 23.0%, *p* < 0.001) [14]. However, Seshasai et al. (2016) found younger ages, males, and white race had a high withdrawal rate than older ages, females, and non-white races [8]. Study differences may be related to gender inequality in treatment and decision-making management [9, 42]. Gender bias in clinical-decision making is still prevalent in many underdeveloped regions and low socioeconomic areas [9, 42]. In a few religions, cultures, societies, races, and ethnicities, women are less privileged than men and have less access to expensive, quality health care such as renal dialysis and transplantation [9, 42]. This difference may also be related to the sample population and differences in defining dialysis withdrawal. Gessert et al. (2013) [14] used all types of dialysis therapies and defined dialysis withdrawal as discontinuation of dialysis due to any reason. However, Seshasai et al. (2016) [8] included only HHD patients and defined dialysis withdrawal as no HHD during a period of > 60 days.

The area of residence was also associated with dialysis withdrawal since residents of small towns and villages have a higher rate of dialysis withdrawal than residents of large cities and towns (26.9% versus 24.3%, *p* < 0.001) [9, 14]. This difference between dialysis rate may be related to the reduced dialysis facilities in small towns and villages compared to cities. Morton et al. (2012) found that the distance to a dialysis centre and the ability to travel are associated with patient choice of dialysis or conservative treatment (discontinuation of dialysis) [43]. Similarly, Elwood et al. (2013) found a higher risk of dialysis withdrawal in patients who had to travel longer than 60 min when compared with patients who had to travel 15 min or less to arrive at the dialysis facility [10]. Authors have also shown that certain marital status such as divorced or widowed and living in nursing homes was also one of the predictors of dialysis withdrawal [31, 44]. However, Birmele et al. (2004) found that living alone or with family or spouse was not a significant predictor of withdrawal [12]. This finding may be due to a small subsample size (*n* = 40) in the withdrawal group. Similarly, some authors have shown that being married, living alone, or divorced was not associated with dialysis withdrawal although the association was significant in an unadjusted analysis [17, 18]. Fissell et al. (2005) found living in a nursing home was significantly associated with dialysis withdrawal in both the adjusted and non-adjusted models, and less than 12 years of education was insignificant in either model [31]. The author also found employment was a significant factor in dialysis withdrawal [31]. All of the studies above defined dialysis withdrawal as discontinuation for any reason and did not provide a subgroup analysis to make further inferences (such as whether the mentioned demographic factors has more influence on any particular reason for dialysis withdrawal). The reasons for the differences between socioeconomic indicators and dialysis withdrawal may be because patients with less education and lower employment status were underprivileged and lacked access to good quality health care as well as the communication skills, transportation, and community support systems required to continue frequent visitation to a dialysis centre for treatment (3–4 times a week for conventional HD) [31, 44].

### Influence of renal disease aetiology on dialysis withdrawal:

There is a scarcity of literature on the association between the aetiology of renal disease with dialysis withdrawal. Ellwood et al. (2013) found that diabetes-induced ESRD and renovascular disease were associated with increased rates of withdrawal (HR = 1.58 [1.37–1.82] and HR = 1.26 [1.06–1.49], respectively) vs. glomerulonephritis [10]. However, Birmele et al. (2004) found that causes of renal diseases such as glomerulopathy, diabetic, interstitial, and vascular nephropathies, and polycystic kidney disease were not associated with dialysis withdrawal [12]. Similarly, another study showed that hypertension, diabetes, and glomerulonephritis were not associated with dialysis withdrawal [19].

These contrasting findings may be attributed to the age of the sample. For example, Ellwood et al. (2013) found that renovascular disease was significantly associated with dialysis withdrawal in patients in the age group < 75 years old [10]. The difference may also be attributed to different definitions of dialysis withdrawal used by different authors. Birmele et al. (2004) define dialysis withdrawal as all types of dialysis discontinuation [12], whereas Ellwood et al. (2013) defined dialysis withdrawal as all types of discontinuation except for recovery patients [10]. Workeneh et al. (2015) defined dialysis withdrawal as the discontinuation of dialysis for several reasons except for transplant and recovery patients [19].

### Influence of health behaviours on dialysis withdrawal

Behaviour risk factors such as smoking, substance abuse, alcohol dependence, and BMI are associated with dialysis withdrawal. Seshasai et al. (2016) showed that smoking and alcohol use were associated with dialysis withdrawal in the HD group (HR = 1.34 [1.01–1.78]) [8]. Similarly, Fissell et al. (2005) showed that alcohol dependence for less than 12 months showed higher odds of dialysis withdrawal in the unadjusted analysis although it was insignificant in the adjusted analysis [31]. Additionally, having a BMI < 18.5 kg/m (HR = 1.37[1.16–1.61]) was associated with increased rates of withdrawal [10]. Patients having a low BMI may have malnutrition and poor health status, thus increasing the odds of dialysis withdrawal as a result of comorbidity worsening and physically deteriorating conditions associated with dialysis [10]. However, categorization of BMI into underweight (< 18.5), healthy (18.5–25), overweight (> 25–30), and obese (> 30) were not associated with PD discontinuation [19]. Similarly, Hazama et al. (2014) found that BMI was not associated with PD withdrawal [16]. The differences in the relationship between dialysis withdrawal and BMI may be attributed to type of dialysis with withdrawal from PD less dependent on BMI when compared with HD [8, 10, 16, 19]. The difference may also be attributed to different causes of dialysis withdrawal selected in each study, such as discontinuation or change of modality [8], technique failure and complications [16], and all types of discontinuation except recovery and transplantation [19].

### Influence of physiology indicators on dialysis withdrawal

Blood and serum markers such as serum albumin, creatinine and dialysate vascular endothelial growth factor are associated with dialysis withdrawal. Hazama et al. (2014) found lower hemoglobin (≤ 11.2 g/dL) and lower serum albumin (≤ 3.31 g/dL) were associated with PD withdrawal, but creatinine, uric acid, and weekly Kt/v were not [16]. The reason for the insignificant association of some of the variables may be related to the type of dialysis, as that study included only PD patients. Hazama et al. (2014) found higher (> 27 pg/mL) dialysate vascular endothelial growth factor was associated with dialysis withdrawal in PD patients. Dialysate vascular endothelial growth factor is a biomarker produced in the peritoneal tissue of patients undergoing PD and has been used as an independent predictor of serum albumin levels [16]. Some authors have shown that excretion of peritoneal albumin was significantly associated with cardiac diseases, resulting in dialysis withdrawal [45–47]. McDade-Montez et al. (2006) found that serum creatinine and phosphate were associated with withdrawal, but not serum potassium [17]. The relationship between serum phosphate and dialysis withdrawal highlights the importance of the dietary control of phosphorus and the use of phosphate-binding medications during dialysis [17]. The association between serum creatinine and dialysis withdrawal may be explained by many patients on dialysis having lower BMI, or lack of adequate nutrition, and reduced muscle mass along with low serum creatinine [17, 48].

### Influence of comorbidities on dialysis withdrawal

Authors have found that comorbidities such as dementia, diabetes, cerebrovascular diseases, and malignancies are associated with dialysis withdrawal. Addition of comorbidities and their combinations may also be positively associated with withdrawal from dialysis [9]. Patients with chronic conditions such as cancer, dementia, diabetes, hypertension, and cachexia are more likely to withdraw than those with acute conditions such as stroke, infection, angina, heart failure, cellulitis and gangrene and infectious diseases such as hepatitis B and C and neurological complications [8–12, 16–19, 31]. Patients with poor health status at the start of dialysis also have a higher risk of dialysis withdrawal. Chronic diseases gradually deteriorate patient health status, leading to complications that initiate a cascade of health issues. These health issues increase the burden of disease and lead patients to discontinue dialysis treatment [38].

In addition to physical health, pain, an important the quality of life measures, is also a significant predictor of dialysis withdrawal. Authors have shown higher withdrawal rates in patients suffering from chronic pain [49]. Davison (2012) found that almost half of patients (50%) have significant pain at the time of dialysis discontinuation [50]. However, patients with comorbidities have a higher risk of depression, despair, loss of positive attitude, and hopelessness than patients without comorbidities [17, 49]. It is difficult to distinguish and understand the biologic plausibility between pain and depression in relation to dialysis withdrawal [17, 49]. It may be that decisions to discontinue dialysis in patients with comorbid conditions and poor health status is due to depression and not chronic pain or discomfort [17, 49].

### Influence of dialysis indicators on dialysis withdrawal

The relationship between type of dialysis such as HD, HHD, and PD, and dialysis withdrawal is inconsistent. Mizuno et al. (2011) found a higher dialysis withdrawal rate in HD patients than PD patients [11]. Chan et al. (2012) found significant effects of PD on dialysis withdrawal in both unadjusted and adjusted models [9]. These differences in findings between studies may be explained by general health status, disease burden, and comorbidities at the start of dialysis [12].

Peritoneal dialysis is mostly performed at home in patients that have more self-control over the treatment and family support to be able to perform routine dialysis [12]. This self-control of dialysis management improves patient’s confidence, active participation in daily activities, and mental health and wellbeing, thus ultimately reducing chances of dialysis withdrawal when compared with in-hospital HD [9, 12]. However, these findings may be attributed to selection bias and confounding factors. Patients having high disease burdens and comorbidities have higher odds of undergoing HD than PD [12]. Poor mental health status has been associated with dialysis withdrawal; therefore, HD patients have higher rates of dialysis withdrawal than PD [49]. The differences in findings may also be due to PD-associated complications. Koc et al. (2011) found that 33% of patient-selected dialysis withdrawal was because of peritonitis (50%) and insufficient PD (50%) [30]. However, few authors found insignificant effects of types of dialysis on dialysis withdrawal [12]. Ellwood et al. (2013) found patients undergoing HD have a higher rate of withdrawal when compared with non-withdrawal, but this association between HD and PD with dialysis withdrawal was insignificant [10]. This inconsistent relationship between the type of dialysis and dialysis withdrawal may be related to the definition of dialysis withdrawal. Koc et al. (2011) define dialysis withdrawal as patient-selected discontinuation [30], while other studies define dialysis withdrawal as the general discontinuation of dialysis for multiple reasons, including technique failure, complications, and preferences of physicians, patients, and relatives [9, 12, 49].

The relationship between duration of dialysis and dialysis withdrawal is inconclusive; McDade-Montez (2006) found an insignificant association for duration of dialysis (in months) between withdrawal and non-withdrawal groups [17]. This finding may be attributed to the small subsample of the dialysis withdrawal group (*n* = 40). Another study showed that duration of dialysis in years was not significantly different between patients who withdrew or continued dialysis [12]. Many dialysis patients have short survival, and exploration of duration of dialysis in years was not an appropriate measure.

### Influence of other individual factors on dialysis withdrawal

There are other individual factors such as late referral to nephrologist, listed for kidney transplantation at the time of dialysis, and community centre versus hospital dialysis, living alone, and quality factors of facility explored by individual studies that have been associated with withdrawal [8–10, 12, 16, 30–32, 51, 52]. There may be different reasons for these associations, such as the characteristics of the study cohort, the type of dialysis therapy included in the study, and the definition of dialysis withdrawal. However, due to the scarcity of studies, these results cannot be explored further. Future studies should investigate these associations to develop stronger correlations.

### Implications for the future research

While authors have explored different factors relating to dialysis withdrawal and revealed differences in dialysis attrition rates, the strength of association for similar factors is inconsistent across various studies. These differences may be due to several reasons. The definition of dialysis withdrawal is not consistent, as different authors have used this concept for multiple reasons and causes. Few authors (*n* = 3) have used a specific definition of dialysis withdrawal. Most authors (*n* = 12) defined dialysis withdrawal as any type of discontinuation, discontinuation, withholding, death, treatment refusal by patients and caregivers, or technique failure [8–12, 16–20, 28, 53]. Discontinuation was defined as no dialysis treatment within a 60 -day period [8]. Withdrawal was defined as either withdrawal from treatment, suicide, accidental death, patient refusal for further treatment, or treatment cessation [9]. Withholding therapy was defined as stopping, not starting, or increasing a life-sustaining intervention. Technique failure was defined as discontinuation of PD for > 6 weeks [17]. Furthermore, few studies have provided exclusion criteria when defining dialysis withdrawal, such as excluding patients with a return of kidney function [10]. Future studies should explore the specific type of dialysis withdrawal or provide a subsequent analysis for each reason or case of dialysis withdrawal to make firm conclusions.

Dialysis withdrawal rate and associated factors are dependent on the type of modality such as HD or PD [8, 9, 12, 19]. Many of the studies have selected either PD or HD patients but not both, making comparisons and inferences difficult to interpret [8, 9]. To determine the role of dialysis modality (HD or PD) in dialysis withdrawal, future studies should explore both modalities in one study setting.

Patients with comorbidities such as diabetes, heart and other chronic debilitating diseases have been shown to be associated with dialysis withdrawal [9]. Poor general health condition due to comorbidities can further reduce dialysis patients’ quality of life, resulting in higher likelihood of dialysis withdrawal than in patients with otherwise good health condition [12, 26, 52]. However, few authors have shown insignificant effects on dialysis withdrawal from diabetes, vascular disease, stroke, cancer, arrhythmias, and lung disease. This difference may be explained by the number of diseases or comorbidities included in the study, duration, severity and types [9, 20, 53]. Many studies have used a small sample or subsample and examined the factors associated with dialysis withdrawal without a priori calculation, resulting in type II error (false negative) [16, 29, 30, 32].

Old ages, females, whites, and those with chronic diseases are associated with dialysis withdrawal [9, 10, 26, 52]. However, few studies showed that demographic factors are not associated with dialysis withdrawal [12, 31]. The geographical setting of the study has also accounted for these differences; factors such as race/ethnicity, preferences of dialysis modality, and whether to withdraw dialysis are sociodemographic dependent [8–12, 16, 17, 26, 31, 51]. Authors should conduct multi-centric and population-based comparative studies to evaluate the influence of demographic factors on dialysis withdrawal.

### Limitations

The review included studies that were published in English language, and studies published in other languages may have been excluded. However, many of our included studies (in English language) were from countries such as China and Japan whose official languages were not English. The main limitation of the review is the scarcity of literature, which limits us to commenting on the similarities and differences between the included studies in relation to the associated factors. However, the primary goal of a scoping review is to systematically search the literature to explore and identify gaps in the selected topic.

## Conclusions

The literature review revealed a scarcity of research on the factors associated with dialysis withdrawal. The findings of the studies are inconsistent and inconclusive due to differences in how dialysis withdrawal is defined among studies, selecting either PD or HD patients as sample population and not both, and selection of multiple comorbidities as predictors. We recommend researchers should conduct studies with a priori calculated sample size and should clearly define the term dialysis withdrawal before exploring the relationships in a sample of HD and PD patients.

## Additional files

Additional file 1: **Table S1.** PubMed Search. (XLSX 8 kb)

Additional file 2: **Table S2.** Embase Search. (XLSX 8 kb)

Additional file 3: **Table S3.** CINAHL Search. (XLSX 9 kb)

## References

[1] Levey AS, Coresh J (2012). Chronic kidney disease. Lancet.

[2] National Institute for Health and Care Excellence. Chronic Kidney Disease: Early Identification and Management of Chronic Kidney Disease in adults in primary and secondary care; 2014 [updated 2015 January; cited 2016 November 27]. Available from: https://www.nice.org.uk/guidance/cg182. https://www.ncbi.nlm.nih.gov/books/NBK51773/. 25340245

[3] Ojo A (2014). Addressing the global burden of chronic kidney disease through clinical and translational research. Trans Am Clin Climatol Assoc.

[4] Steenkamp R, Castledine C, Feest T, Fogarty D (2011). UK renal registry 13th annual report (December 2010): chapter 2: UK RRT prevalence in 2009: national and Centre-specific analyses. Nephron Clin Pract.

[5] Alsuwaida AO, Farag YM, Al Sayyari AA, Mousa D, Alhejaili F, Al-Harbi A (2010). Epidemiology of chronic kidney disease in the Kingdom of Saudi Arabia (SEEK-Saudi investigators) — a pilot study. Saudi J Kidney Dis Transpl.

[6] Arogundade F, Barsoum R (2008). CKD prevention in sub-Saharan Africa: a call for governmental, nongovernmental, and community support. Am J Kidney Dis.

[7] Ontario Renal Network (ORN). Information for Patients: Dialysis. [cited 2016 December 5]. Available from: http://www.renalnetwork.on.ca/info_for_patients/dialysis/#.WFSG7RsrLIU

[8] Seshasai RK, Mitra N, Chaknos CM, Wirtalla C, Negoianu D, Glickman JD (2016). Factors associated with discontinuation of home hemodialysis. Am J Kidney Dis.

[9] Chan HW, Clayton PA, McDonald SP, Agar JW, Jose MD (2012). Risk factors for dialysis withdrawal: an analysis of Australia and New Zealand Dialysis and transplant (ANZADATA) registry, 1999-2008. Clin J Am Soc Nephrol.

[10] Ellwood AD, Jassal SV, Suri RS, Clark WF, Na Y, Moist LM (2013). Early dialysis initiation and rates and timing of withdrawal from dialysis in Canada. Clin J Am Soc Nephrol.

[11] Mizuno M, Ito Y, Tanaka A, Suzuki Y, Kiramatsu H, Watanabe M (2011). Peritonitis is still an important factor for withdrawal from peritoneal dialysis therapy in the Tokai area of Japan. Clin Exp Nephrol.

[12] Birmele B, Francois M, Pengloan J, Francais P, Testou D, Brillet G (2004). Death after withdrawal from dialysis: the most common cause of death in a French dialysis population. Nephrol Dial Transplant.

[13] Murtagh F, Cohen LM, Germain MJ (2007). Dialysis discontinuation: quo Vadis?. Adv in Chronic Kidney Dis.

[14] Gessert CE, Haller IV, Johnson BP (2013). Regional variation in care at the end of life: discontinuation of dialysis. BMC Geriatr.

[15] Chan C, Noble H, Lo S, Kwan TH, Lee S, Sze W (2007). Palliative care for patients with end-stage renal disease: experiences from Hong Kong. Int J Palliat Nurs.

[16] Hazama T, Fukami K, Yamagishi S, Kusumoto T, Sakai K, Adachi T (2014). Dialysate vascular endothelial growth factor is an independent determinant of serus albumin levels and predicts future withdrawal from peritoneal dialysis in uremic patients. Ther Apher Dial.

[17] McDade-Montez EA, Christensen AJ, Cvengros JA, Lawton WJ (2006). The role of depression symptoms in dialysis withdrawal. Health Psychol.

[18] Remon-Rodriguez C, Quiros-Ganga P, Portoles-Perez J, Gomez-Roldan C, Miguel-Carrasco A, Borras-Sans M (2014). Results of the cooperative study of Spanish peritoneal dialysis registries: analysis of 12 years of follow-up. Nefrologia.

[19] Workeneh B, Guffey D, Minard CG, Mitch WE (2015). Causes for withdrawal in an urban peritoneal Dialysis program. Int J Nephrol.

[20] Moist LM, Bragg-Gresham JL, Pisoni RL, Saran R, Akiba T, Jacobson SH (2008). Travel time to dialysis as a predictor of health-related quality of life, adherence, and mortality: the Dialysis outcomes and practice patterns study (DOPPS). Am J Kidney Dis.

[21] Arksay H, O’Malley L (2005). Scoping studies: towards a methodological framework. Int J Soc Res Methodol.

[22] Brien SE, Lorenzetti DL, Lewis S, Kennedy J, Ghali WA (2010). Overview of a formal scoping review on health system report cards. Implement Sci.

[23] Lambert H (2006). Accounting for EBM: notions of evidence in medicine. Soc Sci Med.

[24] Ashuntantang G, Osafo C, Olowu WA, Arogundade F, Niang A, Porter J (2017). Outcomes in adults and children with end-stage kidney disease requiring dialysis in sub-Saharan Africa: a systematic review. Lancet Glob Health.

[25] Noble H, Meyer J, Bridges J, Kelly D, Johnson B (2008). Patient experience of dialysis refusal or withdrawal—a review of the literature. J Ren Care.

[26] O’Connor NR, Kumar P (2012). Conservative Management of end-Stage Renal Disease without Dialysis: a systematic review. J Palliat Med.

[27] Picolli GB, Guzzo G, Vigotti FN, Scognamiglio S, Consiglio V, Aroasio S (2014). Chronic dialysis discontinuation: a systematic narrative review of the literature in the new millennium. Int J Artif Organs.

[28] Sandelowski M, Barroso J (2003). Classifying the findings in qualitative studies. Qual Health Res.

[29] Ashbey M, Hoog C, Kellehear A, Gerr PG, Brooks D, Nicholls K (2005). Renal dialysis abatement: lessons from a social study. Palliat Med.

[30] Koc Y, Unsal A, Ahbap E, Sakacı T, Yilmaz M (2011). Clinical outcome of diabetic peritoneal dialysis patients and evaluation of factors affecting mortality. J Ren Care.

[31] Fissell RB, Bragg-Gresham JL, Lopes AA, Cruz JM, Fukuhara S, Asano Y (2005). Factors associated with “do not resuscitate” orders and rates of withdrawal from hemodialysis in the international DOPSS. Kidney Int.

[32] Urban AK, Brennan F (2013). Patients who withdraw from dialysis in a Sydney Centre with palliative care support: who, why, and how do our patients die?. Prog Palliat Care.

[33] Findlay MD, Donaldson K, Doyle A, Fox JG, Khan I, McDonald J (2016). Factors influencing withdrawal from dialysis: a national registry study. Nephrol Dial Transplant.

[34] Jassal SV, Watson D (2009). Dialysis in late life: benefit or burden. Clin J Am Soc Nephrol.

[35] Bloembergen WE, Port FK, Mauger EA, Wolfe RA (1994). Causes of death in Dialysis patients: racial and gender differences. J Am Soc Nephrol.

[36] Leggat JE, Bloembergen WE, Levine G, Hulbert-Shearon TE, Port FK (1997). An analysis of risk factors for withdrawal from dialysis before death. J Am Soc Nephrol.

[37] Leggat JE, Swartz RD, Port FK (1997). Withdrawal from dialysis: a review with an emphasis on the black experience. Adv Ren Replace Ther.

[38] Neu S, Kjellstarnd CM (1986). Stopping long-term dialysis. An empirical study of withdrawal of life supporting treatment. N Engl J Med.

[39] Port FK, Wolfe RA, Hawthorne VM, Ferguson CW (1989). Discontinuation of dialysis therapy as a cause of death. Am J Nephrol.

[40] Murray AM, Arko C, Chen S-C, Gilbertson DT, Moss AH (2006). Use of hospice in the United States Dialysis population. Clin J Am Soc Nephrol.

[41] Patel SS, Shah VS, Peterson RA, Kimmel PL (2002). Psychosocial variables, quality of life, and religious beliefs in ESRD patients treated with hemodialysis. Am J Kidney Dis.

[42] Vlassoff C (2007). Gender differences in determinants and consequences of health and illness. J Health Popul Nutr.

[43] Morton RL, Snelling P, Webster AC, Rose J, Masterson R, Johnson DW (2012). Factors influencing patient choice of dialysis versus conservative care to treat end-stage kidney disease. CMAJ.

[44] Shen JL, Mitani AA, Saxena AB, Goldstein BA, Winkelmayer WC (2013). Determinants of peritoneal dialysis technique failure in incident us patients. Perit Dial Int.

[45] Heaf JG, Sarac S, Afzal S (2005). A high peritoneal large pore fluid flux causes hypoalbuminaemia and is a risk factor for death in peritoneal dialysis patients. Nephrol Dial Transplant.

[46] Nakamoto H, Imai H, Kawanishi H, Nakamoto M, Minakuchi J, Kumon S (2002). Effect of diabetes on peritoneal function assessed by personal dialysis capacity test in patients undergoing CAPD. Am J Kidney Dis.

[47] Szeto CC, Chow KM, Lam CW, Cheung R, Kwan BC, Chung KY (2005). Peritoneal albumin excretion is a strong predictor of cardiovascular events in peritoneal dialysis patients: a prospective cohort study. Perit Dial Int.

[48] Bircher G, Johnson R, Feehally J (2000). Nutrition in chronic renal failure. Comprehensive clinical nephrology (pp. 75.1–75.8).

[49] Davison SN, Jhnagri GS (2005). The impact of chronic pain on depression, sleep, and the desire to withdraw from dialysis in hemodialysis patients. J Pain Symptom Manag.

[50] Davison SN (2012). The ethics of end-of-life Care for Patients with ESRD. Clin J Am Soc Nephrol.

[51] Foley RN, Collins AJ (2007). End stage renal disease in the United States: an update from the United States renal data system. J Am Soc Nephrol.

[52] Himmelfarb J, Ikizler TA (2010). Hemodialysis. N Engl J Med.

[53] Quinton W, Dillard D, Scribner BH (1960). Cannulation of blood vessels for prolonged hemodialysis. Trans Am Soc Artif Intern Organs.

